# The Calvin Cycle Inevitably Produces Sugar-Derived Reactive Carbonyl Methylglyoxal During Photosynthesis: A Potential Cause of Plant Diabetes

**DOI:** 10.1093/pcp/pcu007

**Published:** 2014-01-30

**Authors:** Daisuke Takagi, Hironori Inoue, Mizue Odawara, Ginga Shimakawa, Chikahiro Miyake

**Affiliations:** ^1^Department of Biological and Environmental Science, Faculty of Agriculture, Graduate School of Agricultural Science, Kobe University, 1-1 Rokkodai, Nada, Kobe, 657-8501 Japan; ^2^CREST, JST, 7 Gobancho, Chiyoda-ku, Tokyo, 102-0076 Japan; ^3^These authors contributed equally to this work.

**Keywords:** Advanced glycation end-products, Methylglyoxal, Photosynthesis, Triose phosphate isomerase

## Abstract

Sugar-derived reactive carbonyls (RCs), including methylglyoxal (MG), are aggressive by-products of oxidative stress known to impair the functions of multiple proteins. These advanced glycation end-products accumulate in patients with diabetes mellitus and cause major complications, including arteriosclerosis and cardiac insufficiency. In the glycolytic pathway, the equilibration reactions between dihydroxyacetone phosphate and glyceraldehyde 3-phosphate (GAP) have recently been shown to generate MG as a by-product. Because plants produce vast amounts of sugars and support the same reaction in the Calvin cycle, we hypothesized that MG also accumulates in chloroplasts. Incubating isolated chloroplasts with excess 3-phosphoglycerate (3-PGA) as the GAP precursor drove the equilibration reaction toward MG production. The rate of oxygen (O_2_) evolution was used as an index of 3-PGA-mediated photosynthesis. The 3-PGA- and time-dependent accumulation of MG in chloroplasts was confirmed by HPLC. In addition, MG production increased with an increase in light intensity. We also observed a positive linear relationship between the rates of MG production and O_2_ evolution (*R* = 0.88; *P* < 0.0001). These data provide evidence that MG is produced by the Calvin cycle and that sugar-derived RC production is inevitable during photosynthesis. Furthermore, we found that MG production is enhanced under high-CO_2_ conditions in illuminated wheat leaves.

## Introduction

Glycolysis is the most important metabolic pathway for producing cellular energy sources (i.e. NADPH and ATP) from sugars in heterotrophs, including humans. However, this pathway inevitably generates reactive carbonyls (RCs), including methylglyoxal (MG), glyoxal (GLO) and 3-deoxyglucosone (3-DG), as by-products. First, triose phosphate isomerase (TPI) catalyzes the equilibration reaction between dihydroxyacetone phosphate (DHAP) and glyceraldehyde 3-phosphate (GAP), producing MG and GLO as by-products (Phillips and Thornalley 1993). Secondly, DHAP non-enzymatically reacts with GAP to produce MG and GLO (Phillips and Thornalley 1993). Thirdly, the aldehyde group of sugars reacts with free amino acids or amino acid groups in proteins to produce a Schiff base, which is converted to an RC by Amadori rearrangement (Vistoli et al. 2013). Fourthly, sugars containing an aldehyde group undergo auto-oxidation and degradation to produce MG, GLO and 3-DG (Vistoli et al. 2013). Fifthly, sugar auto-oxidation produces superoxide radicals (O^−^_2_) (Medina-Navarro et al. 2003), which are rapidly converted into hydrogen peroxide (H_2_O_2_) and oxygen (O_2_) by superoxide dismutase. The Fenton reaction catalyzes the conversion of these products into the most potent reactive oxygen species (ROS), hydroxyl radicals (· OH), using transition metals such as iron (Fe) and copper (Cu) (Stadtman and Levine 2003). The degradation of sugars by · OH produces RCs as by-products. Therefore, RCs are inevitably generated by sugar metabolism in the glycolytic pathway essential for cellular energy production in heterotrophs.

Sugar-derived RCs react aggressively with all major cell components, including DNA, proteins and lipids, resulting in reduced or aberrant physiological functions. For example, the interaction between MG, GLO or 3-DG and amino acids (arginine, lysine or histidine) produces glycated amino acids in proteins: carboxylmethyl-lysine, carboxylethyl-lysine, GLO-arginine, GLO-histidine and 3DG-histidine (Thornalley et al. 1999). They cross-link to form dimers (GLO-derived lysine dimer, MG-derived lysine dimer and 3DG-derived lysine dimer) (Aldini et al. 2013, Vistoli et al. 2013). These RC-modified proteins, which are called advanced glycation end-products (AGEs), have lost their physiological function. These AGEs are found in excess in patients with diabetes mellitus, and they are associated with major complications, including retinopathy, nephropathy, neuropathy, arteriosclerosis and cardiac insufficiency (Jaganjac et al. 2013).

Vertebrates, including humans, possess defense mechanisms against these sugar-derived RCs, which suppress AGE accumulation within cells. Aldo–keto reductase (AKR), aldehyde dehydrogenase (ALDH) and the glyoxalase (GLX) system together support RC detoxification (Aldini et al. 2013). AKR reduces the levels of RCs to those of corresponding acetols using NADPH, and ALDH oxidizes RCs using NAD^+^. The GLX system neutralizes MG using two enzymes (GLXI and GLXII) and glutathione (GSH), and GLX specifically scavenges MG. In the GLX system, GSH first reacts with MG to form hemithio-acetal (HA) non-enzymatically. HA is transformed to *S*-lactoylglutathione (SLG) by GLXI, and SLG is then transformed to lactic acid by GLXII, leading to the regeneration of GSH.

How do photosynthetic autotrophs such as plants obliterate the threat of RC attacks? Why do plants not suffer from diabetes? Plant leaves accumulate high concentrations of sugars (approximately hundreds of micromoles) in their cells during photosynthesis (Qiu et al. 2008), indicating that the threat of sugar-derived RCs and ROS would be higher in autotrophs than in heterotrophs. Qiu et al. (2008) reported that carbonylated proteins due to RCs and ROS accumulate in the leaves of plants exposed to high CO_2_ concentrations. They initially did not expect accumulation of the modified proteins under high CO_2_ concentrations when photosynthesis was stimulated because enhanced photosynthesis suppresses the production of ROS in chloroplasts (Asada 1999). Photoreduction of O_2_ to O^−^_2_ in PSI of the thylakoid membranes is inevitable in illuminated chloroplasts (Asada 1999). Photoreduction of O_2_ competes with that of NADP^+^ at PSI. Following this, photosynthesis suppresses the production of ROS under high CO_2_ concentrations, indicating that the oxidative modification of proteins by RCs and ROS is suppressed under high CO_2_ concentrations. Bechtold et al. (2009) first detected AGEs in plant leaves. The concentrations of many AGEs fluctuate during day/night cycles. Based on these results, we propose the hypothesis that carbon metabolism in plant leaves, which photosynthesize and fix CO_2_ to sugars, always accompanies RC production. In particular, the risk of suffering from diabetes mellitus is high in environments where photosynthesis is enhanced in high light or high CO_2_ concentrations even in organisms that perform photosynthesis.

Higher plants possess RC detoxification enzymes, similar to vertebrates (Paulus et al. 1993, Mundree et al. 2000, Saito et al. 2013). As observed in humans, AKR functions by scavenging MG in higher plants. AKR is found in a family comprising 14 subfamilies, and the AKR4C subfamily scavenges MG (Saito et al. 2013). Furthermore, other RC detoxification enzymes have been proposed to function in stressful environments. Gene expression in the GLX system and AKR subfamilies is enhanced under abiotic stressors such as salinity, osmotic pressure and heat (Singla-Pareek et al. 2003). Activity of the AKR subfamily increases under drought conditions (Simpson et al. 2009). Furthermore, genetically modified plants that have higher AKR activity show tolerance against these stressful conditions (Sunkar et al. 2003). We observed increases in AKR4C subfamily gene expression in the leaves of plants exposed to high CO_2_ concentrations and high light environments (Saito et al. 2013). These observations support the hypothesis that the risk of diabetes mellitus increases when photosynthesis is enhanced.

In the present study, we elucidated the MG production mechanism during photosynthesis and the physiological function of photosynthetic activity on MG production. We used MG as an index of RC production during carbon fixation by the light-dependent Calvin cycle in isolated chloroplasts. MG is a glycolytic by-product produced during the equilibration reaction between DHAP and GAP catalyzed by TPI (Richard 1991). Because this enzyme regulates the same reactions in the Calvin cycle, we expected MG to be generated as a by-product of CO_2_ fixation into sugars in plants. We initiated 3-phosphoglycerate (3-PGA)-dependent O_2_ evolution in isolated chloroplasts. 3-PGA is metabolized to GAP, which is sequentially catalyzed by PGA kinase and GAP dehydrogenase (GAPDH). GAP is equilibrated with DHAP, which is catalyzed by TPI. We observed MG production only in the presence of 3-PGA in illuminated chloroplasts. The MG production rate was dependent on light intensity. MG production showed a positive linear relationship with 3-PGA-dependent O_2_ evolution. Furthermore, we found enhanced MG production under high [CO_2_] in illuminated wheat leaves. These results demonstrate that enhanced MG production is inevitable for stimulating photosynthesis under high CO_2_ concentrations.

## Results

### PGA-dependent O_2_ evolution accompanied the production of MG and GLO in intact chloroplasts

The mechanism of sugar-derived RC production during photosynthesis was elucidated by studying MG/GLO metabolism. MG and GLO are produced during glycolysis by enzymatic or non-enzymatic equilibrium reactions between DHAP and GAP (Thornalley 2006). Because these reactions are also a part of the Calvin cycle in chloroplasts, we hypothesized that GAP production would stimulate the equilibration reaction between GAP and DHAP catalyzed by TPI and that the production of MG and GLO would be enhanced in chloroplasts.

The production of GAP by the Calvin cycle was induced by adding 3-PGA to an intact chloroplast suspension, which enters the stroma through the phosphate–triose phosphate–phosphoglycerate translocator (Flügge and Heldt 1984). 3-PGA is sequentially converted to 1,3-bisphosphoglycerate and then to GAP by PGA kinase and GAP dehydrogenase, respectively. These reactions consume NADPH and ATP, which are regenerated by photosynthetic linear electron flow. Because the addition of 3-PGA to actinic red-illuminated chloroplasts induces O_2_ evolution, the rate of O_2_ evolution reflects the GAP production rate in chloroplasts (Takagi et al. 2012).

**Fig. 1:**
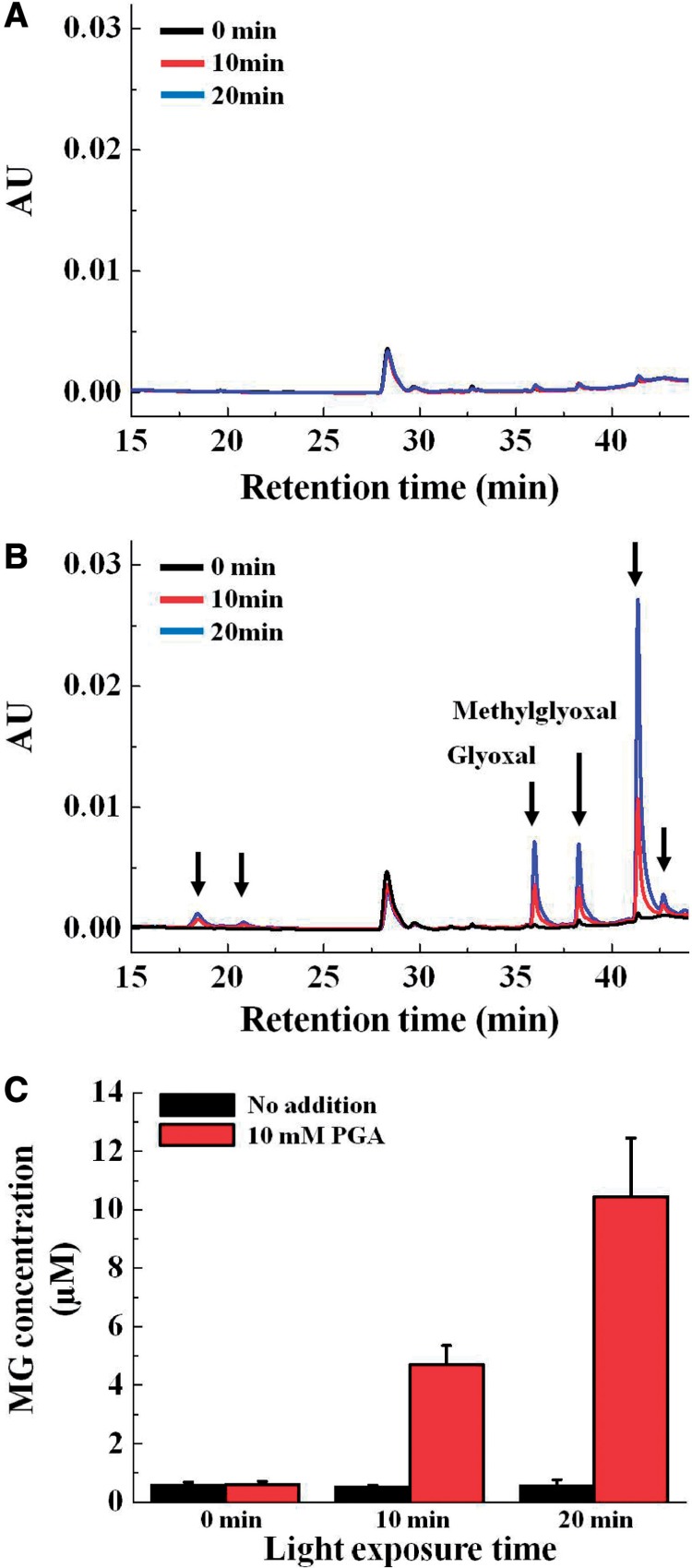
Chromatograms of *o*-phenylenediamine (OPD) derivatives of sugar-derived reactive carbonyls (RCs). 3-Phosphoglycerate (3-PGA) was absent (A) and present (B) in the reaction mixtures. Reaction mixtures (1 ml) that contained chloroplasts (40 µg of Chl) in the absence and presence of 10 mM 3-PGA were illuminated in red light (>640 nm, 400 µmol photons m^−2^ s^−1^) at 25°C. Typical chromatograms of sugar-derived RCs are shown. AU indicates relative absorbance units at 312 nm. The three lines indicate different light exposure times (black, 0 min; red, 10 min; and blue, 20 min). Arrows indicate sugar-derived RCs, the levels of which increased in the presence of 3-PGA. Glyoxal (GLO) and methyglyoxal (MG) were identified from the retention times of the commercially purchased compounds. (C) MG was quantified at the indicated time after illumination in the absence (black bars) or presence (red bars) of 3-PGA. HPLC of sugar-derived RCs is described in the Materials and Methods section. Values are expressed as means ± standard deviations of three independent experiments.

Dicarbonyl compounds (MG, GLO and 3-DG) react with *o*-phenylenediamine (OPD) to produce quinoxaline derivatives (Mavric et al. 2008). The OPD-derived compounds were separated by HPLC and quantitatively detected by spectrophotometry (Mavric et al. 2008). The illumination of intact chloroplasts (400 µmol photons m^−2^ s^−1^) in the absence of 3-PGA did not induce the production of sugar-derived RCs such as MG, GLO and 3-DG (Fig. 1a; Supplementary Fig. S1) or O_2_ evolution (data not shown). Incubating intact chloroplasts with 10 mM PGA in the dark did not induce the production of the sugar-derived RCs, MG (Supplementary Fig. S2) and GLO (data not shown). In contrast, the addition of 3-PGA to intact chloroplasts under illumination induced the production of MG and GLO (Fig. 1b; Supplementary Fig. S1) and induced O_2_ evolution (data not shown). The amounts of these RCs increased linearly with prolonged illumination (Fig. 1c). No RC was observed under dark conditions even in the presence of 3-PGA (data not shown). Furthermore, we confirmed that 3-PGA did not react with OPD to produce the quinoxaline form (data not shown). In addition, we found four peaks (retention times: 17, 21, 41 and 42 min) in the chromatography profile that were not identified in the present study (Fig. 1b). As a result, we found six compounds that accumulated in the presence of 3-PGA. These results indicate that the turnover of the TPI reaction in the Calvin cycle produced MG and GLO, similar to that observed in the glycolytic pathway.

**Fig. 2:**
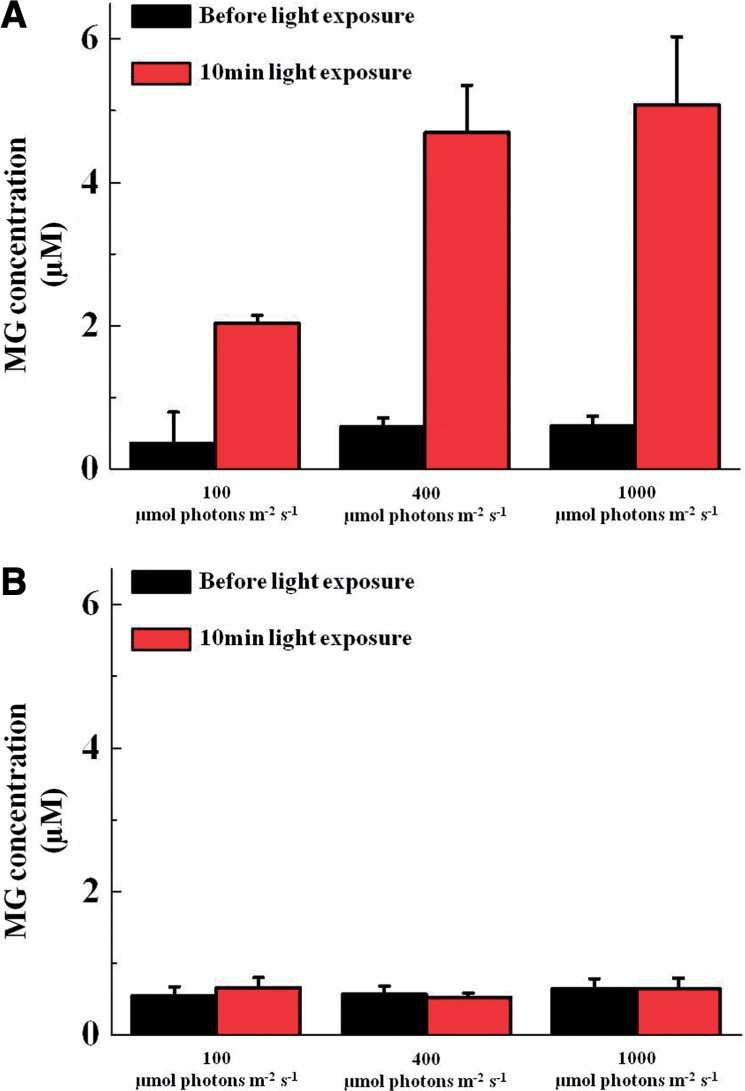
Light dependency of methyglyoxal (MG) production in chloroplasts. The reaction mixtures (1 ml) containing chloroplasts (40 µg of Chl) in the presence (A) and absence (B) of 10 mM 3-phosphoglycerate (3-PGA) were illuminated at the indicated intensity of red light for 10 min. Black bar, in the dark; red bars, exposure at the indicated light intensity. Values are expressed as means ± SD of three independent experiments.

Following this, we tested the effect of light intensity on MG production in intact chloroplasts (Fig. 2a). The MG content increased with the increase in light intensity in the presence of 3-PGA. In contrast, this increase was not observed in the absence of 3-PGA (Fig. 2b). We also observed that the increase in light intensity enhanced GLO production (Supplementary Fig. S1).

### Relationship between photosynthetic activity and MG production in intact chloroplasts

**Fig. 3:**
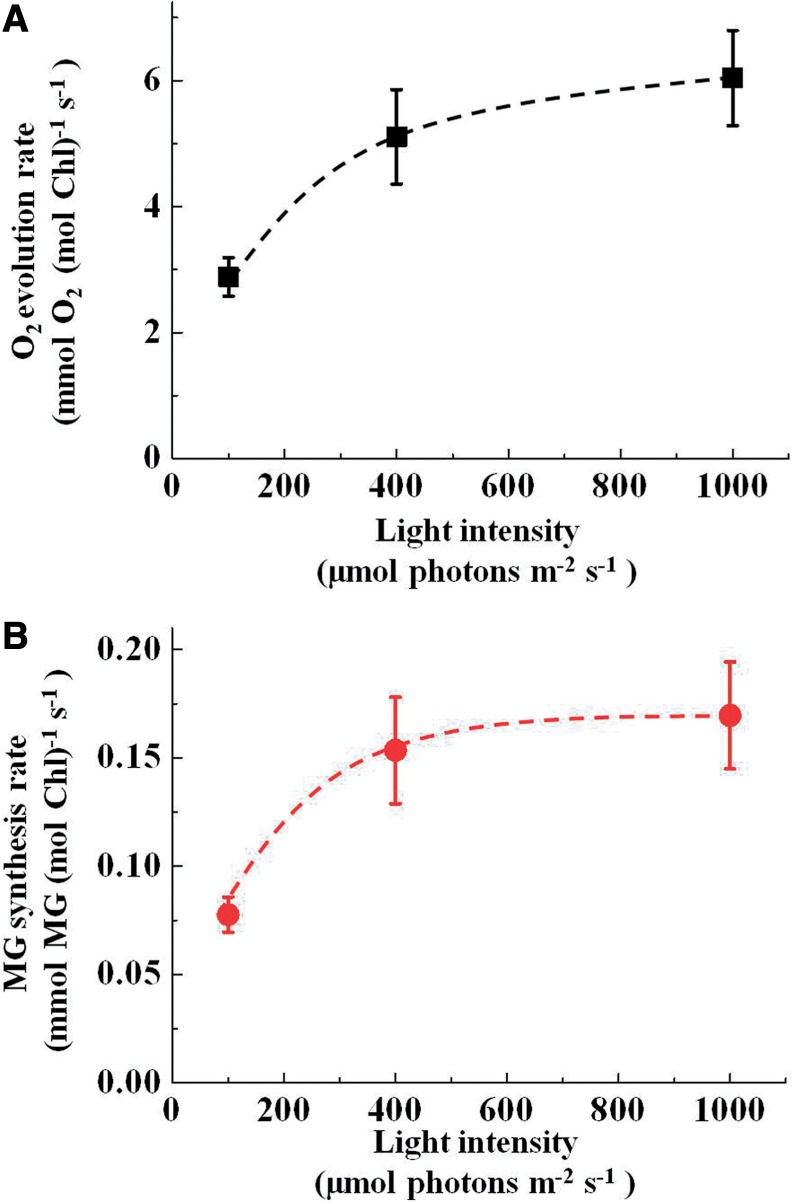
Dependence of the O_2_ evolution rate (A) and methyglyoxal (MG) synthesis rate (B) on the intensity of red light in chloroplasts. The reaction mixture (1 ml) containing 40 µg of chloroplasts and 10 mM 3-phosphoglycerate (3-PGA) was illuminated at the indicated light intensity. The O_2_ evolution rate was calculated from the steady-state increase in [O_2_] at 10 min. The MG synthesis rate was calculated from the concentration of MG at 10 min. Values are expressed as means ± SE of five independent experiments.

**Fig. 4:**
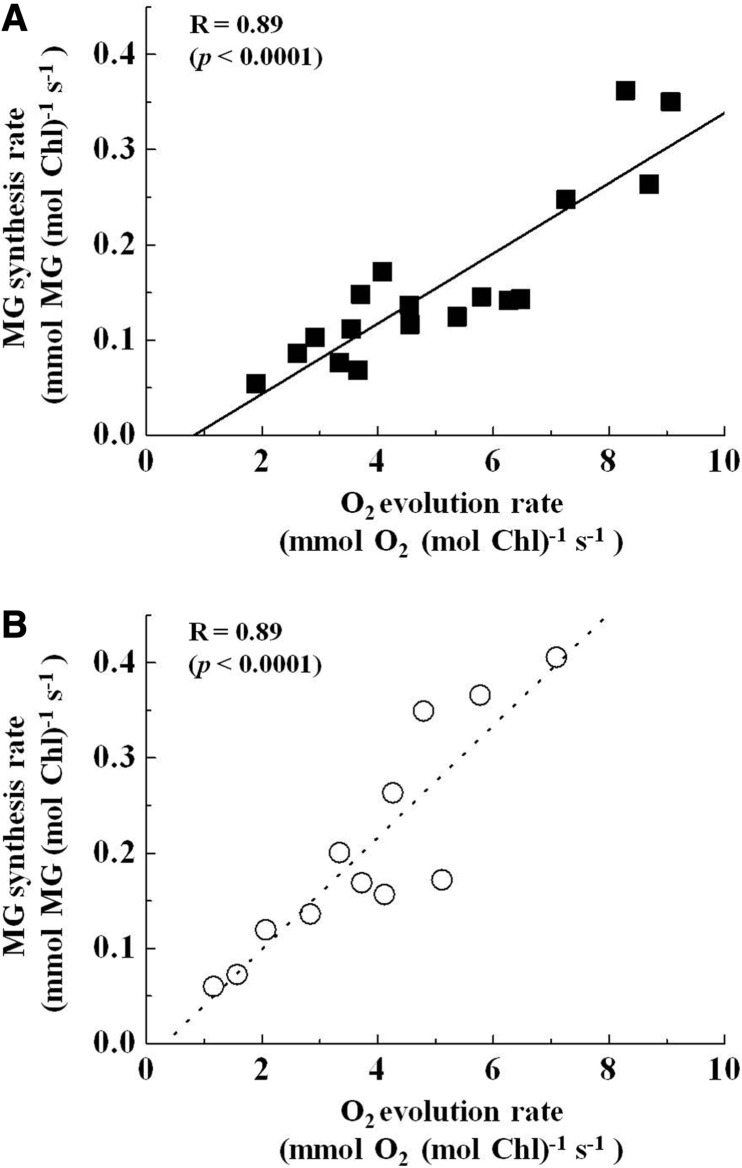
The relationship between the O_2_ evolution rate and methyglyoxal (MG) synthesis rate. The O_2_ evolution rate and MG synthesis rate were obtained from Fig. 3. (A) The O_2_ evolution rates were plotted against the MG synthesis rates. These rates were estimated at 25°C. The positive linear line was the fitted line with the linear regression model. (B) Both the O_2_ evolution rates and MG synthesis rates were estimated at 35°C (data not shown), and the O_2_ evolution rates were plotted against the MG synthesis rates. The positive linear dotted line is the line fitted with the linear regression model. The correlation coefficient (*R*) and *P*-value are shown. Values were obtained from five independent experiments.

We calculated the rates of O_2_ evolution and MG production and plotted them against light intensity (Fig. 3). Both O_2_ evolution and MG production rates increased with an increase in light intensity at 25°C. MG production rates corresponded to 3% of the O_2_ evolution rate at 100 µmol photons m^−2^ s^−1^ and a saturated intensity of 1,000 µmol photons m^−2^ s^−1^. Furthermore, a positive linear relationship was observed between the rates of MG production and O_2_ evolution (Fig. 4a).

Finally, we tested the impact of temperature on MG production during photosynthesis. We plotted the MG production rate against the O_2_ evolution rate at 35°C (Fig. 4b). The MG production rate showed a positive linear relationship with the O_2_ evolution rate, similar to that observed at 25°C. However, the slope of the positive line at 35°C was larger than that at 25°C. Therefore, these data show that high temperature enhances MG production in chloroplasts, which could be due to an increase in TPI activity. As a result, the production rate of the MG by-product increased.

### High [CO_2_] stimulated photosynthesis with enhanced MG production in wheat leaves

As shown above, the presence of 3-PGA stimulated MG production in intact chloroplasts. This result shows that stimulating the turnover of the Calvin cycle enhances MG production. Following this, we tested whether stimulation of photosynthesis accompanied enhanced MG production in the intact leaves of higher plants. We compared the MG production in illuminated wheat leaves under different CO_2_ concentrations.

**Fig. 5:**
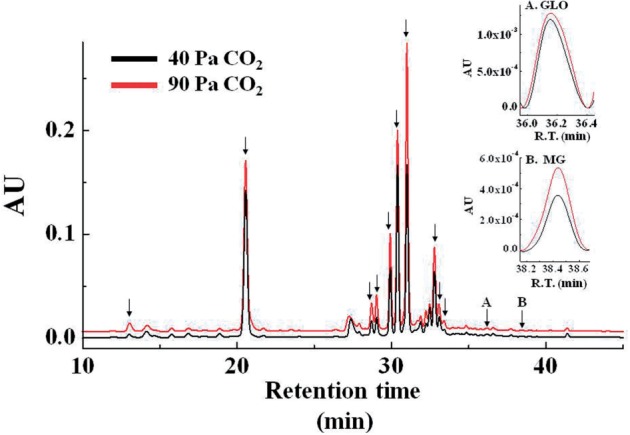
Chromatograms of *o*-phenylenediamine (OPD) derivatives of sugar-derived reactive carbonyls (RCs). Black line, 40 Pa CO_2_; Red line, 90 Pa CO_2_. Typical chromatograms of sugar-derived RCs are shown. AU indicates the relative absorbance units based on leaf area at 312 nm. Arrows indicate sugar-derived RCs, the levels of which increased at 90 Pa CO_2_ (compared with 40 Pa CO_2_). Glyoxal (GLO) and methyglyoxal (MG) were identified from the retention times of the commercially purchased compounds. Insets show typical MG and GLO peaks, which were normalized with the baseline. HPLC of sugar-derived RCs is described in the Materials and Method. R.T., retention time.

We illuminated wheat leaves at 1,060 µmol photons m^−2^ s^−1^, 20% O_2_ and 40 Pa CO_2_ for 1 h. The steady-state rate of photosynthetic CO_2_ fixation was 15 ± 3 µmol CO_2_ m^−2^ s^−1^ (*n* = 3). Following this, we analyzed dicarbonyl compounds in the illuminated leaves (Fig. 5). We observed MG and GLO production in intact wheat leaves (Fig. 5). The concentrations of MG and GLO were 1.9 ± 0.2 µmol m^−2^ (*n* = 3) and 117 ± 19 µmol m^−2^ (*n* = 3), respectively (Table 1).

**Table 1:** Effect of an increase in CO_2_ partial pressure on net CO_2_ assimilation rate (*A*) and concentrations of MG and GLO in wheat leaves

Conditions	*A* (µmol CO_2_ m^–2^ s^–1^)	MG (µmol m^–2^)	GLO (µmol m^–2^)
40 Pa CO_2_	15 ± 3	1.9 ± 0.2	117 ± 19
90 Pa CO_2_	21 ± 2	2.8 ± 0.6	150 ± 40

Following this, we illuminated wheat leaves at high [CO_2_] (1,060 µmol photons m^−2^ s^−1^, 20% O_2_ and 90 Pa CO_2_) for 1 h. The steady-state rate of photosynthetic CO_2_ fixation was 21 ± 2 µmol CO_2_ m^−2^ s^−1^ (*n* = 3), which was higher than that at 40 Pa CO_2_. We analyzed dicarbonyl compounds (Fig. 5) and found many peaks in the HPLC chromatograph, some of which were larger than those at 40 Pa CO_2_. These results indicate that stimulation of photosynthesis induces the production of dicarbonyl compounds. MG and GLO concentrations were 2.8 ± 0.3 µmol m^−2^ (*n* = 3) and 150 ± 40 µmol m^−2^ (*n* = 3), which were higher than those at 40 Pa CO_2_. In other words, stimulating photosynthesis at high [CO_2_] increased MG and GLO concentrations by approximately 50% (0.9 µmol m^−2^) and 30% (30 µmol m^−2^), respectively. If all the detected MG was derived from chloroplasts, the increased concentration was estimated to be approximately 150 µM in chloroplasts [200 mg (mg Chl) m^−2^ wheat leaves; 30 µl stroma (mg Chl)^−1^ (Heldt et al. 1973)].

We found many peaks on the HPLC chromatograph, some of which were different from those found in intact chloroplasts. We did not identify these peaks, which remain to be clarified in order to elucidate the physiological function of these dicarbonyl compounds.

## Discussion

We hypothesized that 3-PGA-dependent O_2_ evolution initiates the production of MG in isolated chloroplasts. 3-PGA was metabolized to GAP, which was sequentially catalyzed by PGA kinase and GAPDH, and GAP was equilibrated with DHAP, which was catalyzed by TPI. MG and GLO were detected only in the presence of 3-PGA (Figs. 1, 2). The MG production rate increased with the illumination time and light intensity. MG production showed a positive linear relationship with 3-PGA-dependent photosynthesis (Fig. 4). These results suggest that the production of MG via the Calvin cycle was the same as that observed during glycolysis and that MG and GLO production was inevitable during photosynthesis. Furthermore, we found that MG and GLO production was enhanced under high [CO_2_] in intact leaves. Photosynthetic activity increased at high [CO_2_]. In other words, the stimulated turnover of the Calvin cycle induced the production of the by-products MG and GLO.

We observed the production of other RCs in isolated chloroplasts and intact leaves (Fig. 1). Furthermore, we observed many RCs, some of which were different from those found in intact chloroplasts. The production mechanisms of those RCs remain unknown. MG and GLO produced in the Calvin cycle may induce the production of RCs by affecting metabolism during photosynthesis or may inactivate the enzymes during oxidative attacks on lipid metabolism (Vistoli et al. 2013). Furthermore, the triose phosphate photosynthates, including DHAP and GAP, which were exported from the chloroplasts to the cytosol, would induce MG and GLO production in the cytosol. These dicarbonyls would affect or disrupt cytosolic metabolism, leading to the production of other dicarbonyls detected in leaves.

We evaluated the steady-state concentration of MG in chloroplasts. Chloroplasts show AKR activity (Saito et al. 2011). The *V*_max_ and *K*_m_ values for MG in the AKR reaction of chloroplasts are 3 µmol NAPDH (mg Chl)^−1^ h^−1^ and 5 mM, respectively (Saito et al. 2011). The maximum MG production rate during 3-PGA-dependent O_2_ evolution was approximately 0.6 µmol MG (mg Chl)^−1^ h^−1^ (Fig. 3). The steady-state concentration of MG was estimated to be 1.3 mM. At the steady state, the MG production rate was equal to the scavenging rate by AKR, which followed the Michaelis–Menten-type reaction as follows:

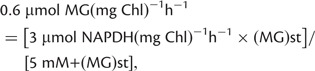

where (MG)st is the steady-state concentration of MG (mM) and was estimated to be 1.3 mM.

During the light phase of photosynthesis, MG catalyzes the photoreduction of O_2_ to O^−^_2_ in the PSI complex (Saito et al. 2011). The *K*_m_ for MG is approximately 0.2 mM. MG may catalyze the photoreduction of O_2_ at PSI during 3-PGA-dependent O_2_ evolution. When MG is univalently reduced, it forms an MG radical (see figure below; Yim et al. 1995) with a mid-point redox potential (E′m) of −330 mV (Kalapos 2008). If this reaction occurs in PSI, the reduced MG presumably donates electrons to O_2_, producing O^−^_2_.


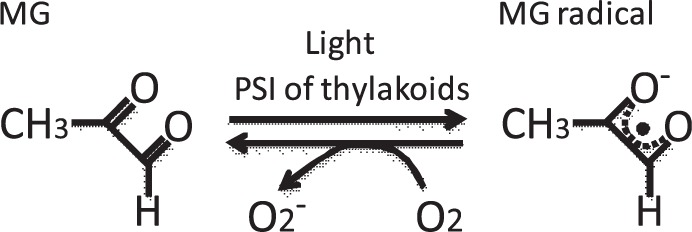


The candidates for electron transport components that catalyze the univalent reduction of MG at PSI are phylloquinones (E′m: −800 mV); F_x_, an interpolypeptide (4Fe–4S cluster) (E′m: −705 mV); F_A_ (E′m: −520 mV) and F_B_ (E′m: −580 mV), which are 4Fe–4S clusters bound to the extrinsic subunit PsaC (Nelson and Yocum 2006); and ferredoxin (E′m: −430 mV). O^−^_2_ is disproportionate to H_2_O_2_ and H_2_O. In other words, the photoreduction of O_2_ at PSI stimulates the production of H_2_O_2_. Furthermore, the accumulation of O^−^_2_ and H_2_O_2_ stimulates the production of ·OH by the transition metal-catalyzed Fenton reaction. ·OH has a higher redox potential than O^−^_2_ and H_2_O_2_, and can fragment DNA, proteins and lipids (Asada and Takahashi 1987). In particular, the stimulated production of ·OH in chloroplast fragments ribulose 1,5-bisphosphate carboxylase/oxygenase and glutamine synthase, and these enzymes degrade oxidatively (Fucci et al. 1983, Ishida et al. 1997, Ishida et al. 1998). In addition, ·OH decomposes the PSI complex at a low temperature (Sonoike et al. 1996, Tjus et al. 2001). These oxidative attacks by ·OH would be one of the reasons why the carbonylated proteins accumulate in plants cultivated at high CO_2_ concentrations (Qiu et al. 2008).

### Conclusion

In the present study, we first showed that MG and GLO production in chloroplasts depended on light and 3-PGA, indicating that MG and GLO were produced by the Calvin cycle during photosynthesis. Similar to glycolysis, MG and GLO would be generated as by-products of the TPI reaction in the Calvin cycle. Therefore, if photosynthesis is enhanced by high [CO_2_] levels, the metabolic turnover of the Calvin cycle accelerates, resulting in enhanced MG and GLO production. In fact, we observed enhanced MG and GLO production at high [CO_2_]. We also observed higher rates of MG production at 35°C than at 25°C. As global warming proceeds, the temperature on the earth will continue to increase. This will increase TPI activity, resulting in an increased production rate of MG. Research on the production mechanisms of sugar-derived RCs is indispensable for elucidating the physiological functions of RCs in crops exposed to a global warming environment.

## Materials and Methods

### Plant materials and growth conditions

Wheat (*Triticum aestivum* L. cv. Mironovskaya 808) was grown in soil (commercial peat-based compost) for 4–5 weeks under 14 h light (25°C)/10 h dark (20°C) and 500 µmol photons m^−2^ s^−1^.

### Isolation of intact chloroplasts from spinach leaves

Spinach leaves from a local market were processed to isolate intact chloroplasts, as described previously (Takagi et al. 2012). The chloroplasts were suspended in a reaction buffer containing 50 mM HEPES–KOH (pH 7.4), 0.33 M sorbitol, 10 mM NaCl, 1 mM MgCl_2_, 2 mM EDTA, 0.5 mM KH_2_PO_4_ and 1 mM ascorbate. This chloroplast suspension was purified on a Percoll gradient. The purified chloroplasts were approximately 80–90% intact based on the ferricyanide method (Heber and Santarius 1970). The Chl content was determined using the method of Arnon (1949).

### O_2_ exchange in isolated chloroplasts

The rate of 3-PGA-dependent O_2_ evolution was measured in isolated chloroplasts, as described previously. In brief, the chloroplast suspension was placed in different O_2_ electrode cuvettes (DW2/2; Hansatech Ltd.) with actinic red light (>640 nm) in the absence or presence of 3-PGA. At specific time intervals, aliquots of the chloroplast solution were collected for analysis of sugar-derived RCs. Temperature-controlled water was circulated through the water jacket during the assay and was maintained at 25 or 35°C.

### Gas-exchange analysis

The rates of CO_2_ and H_2_O vapor exchange were measured with an open gas-exchange system using a temperature-controlled chamber. The system is detailed in Makino et al. (1988). Differences in the partial pressures of CO_2_ and H_2_O entering and leaving the chamber were measured with an IRGA (LI-7000, Li-COR). Gas with the indicated mixture of pure O_2_ and CO_2_ was prepared by mixing 20.1% (v/v) in 79.9% (v/v) N_2_ and 1% (v/v) CO_2_ in 99% (v/v) N_2_ using a mass-flow controller (Kofloc model 1203; Kojima Instruments Co.). The mixture of gases was saturated with water vapor at 13.5 ± 0.1°C. The photosynthetic photon flux density at the position of the leaf in the chamber was adjusted to 1,060 µmol photons m^−2^ s^−1^. The leaf temperature was controlled at 25°C. Gas-exchange parameters were calculated according to the equations in von Caemmerer and Farquhar (1981). The leaf in the chamber was rapidly removed and frozen in the liquid N_2_ solution until RC analysis. Freezing took approximately 1 s.

### Analysis of sugar-derived RCs

The sugar-derived RCs (1,2-dicarbonyls) generated by 3-PGA-dependent O_2_ evolution were identified as the corresponding quinoxalines after derivatization with OPD, as described previously (Mavric et al. 2008). Aliquots of isolated intact chloroplasts (1 ml) were mixed with 1 ml of sodium phosphate buffer [50 mM NaH_2_PO_4_, 0.2% (w/v) OPD, pH 6.5] and incubated at room temperature for 12 h in the absence of light. During this incubation, the intact chloroplasts were disrupted by hypotonic shock and the released sugar-derived RCs reacted with OPD to produce quinoxaline derivatives. Following this, the suspension was centrifuged (10,000 × *g*; 10 min), and the supernatant was filtered through a 0.45 µm membrane. The quinoxaline derivatives were identified by HPLC. In brief, 40 µl of samples were injected, and the elution was performed at a flow rate of 0.8 ml min^–1^. Solvent A contained 0.150% (v/v) acetic acid, and solvent B comprised 80% (v/v) aqueous methanol containing 0.150% (v/v) acetic acid. The gradient started with 20% solvent B, followed by a linear increase to 40% solvent B over 20 min and to 100% solvent B within 15 min. After the elution with 100% solvent B for 5 min, a linear decrease to 20% solvent B over 7 min, and then an equilibration with 20% solvent B for 30 min. The analytical column was Knauer Eurospher 100 C18 (5 μm; 4.6 × 250 mm). The wavelength was set to 312 nm. External calibration was performed using a linear calibration curve with MG (0.1–300 µg ml^–1^) purchased from Nacalai Tesque.

The sugar-derived RCs (1,2-dicarbonyls) generated during the photosynthesis of intact leaves were identified as described above. Frozen leaves were mixed in a chilled mortar and pestle in sodium phosphate buffer (1 ml) [50 mM NaH_2_PO_4_, 0.2% (w/v) OPD, pH 6.5] and further incubated at room temperature for 12 h in the absence of light. Following this, the suspension was centrifuged (10,000 × *g*; 10 min), and the supernatant was filtered through a 0.45 µm membrane. The quinoxaline derivatives were identified by HPLC, as described above.

## Supplementary data

Supplementary data are available at PCP online.

## Funding

This study was supported by the Japan Society for the Promotion of Science [Scientific Research Grant No. 21570041 to C.M.]; the Ministry of Education, Culture, Sports, Science, and Technology in Japan [Scientific Research on Innovative Area No. 22114512 to C.M.].

## Supplementary Material

Supplementary Data

## Abbreviations

AGE: advanced glycation end-product
AKR: aldo–keto reductase
3-DG: 3-deoxyglucosone
DHAP: dihydroxyacetone phosphate
GAP: glyceraldehydes 3-phosphate
GLO: glyoxal
GLX: glyoxylase
MG: methylglyoxal
OPD: *o*-phenylenediamine
3-PGA: 3-phosphoglycerate
RC: reactive carbonyl
ROS: reactive oxygen species
TPI: triose phosphate isomerase
