# Supplementary material for: The Calvin Cycle Inevitably Produces Sugar-Derived Reactive Carbonyl Methylglyoxal During Photosynthesis: A Potential Cause of Plant Diabetes
Source: Plant Cell Physiol. 2014 Jan 30;55(2):333–40. doi: 10.1093/pcp/pcu007 (PMC3913449; doi:10.1093/pcp/pcu007)
Supplement: Supplementary Data [file supp_55_2_333__index.html]

The Calvin Cycle Inevitably Produces Sugar-Derived Reactive Carbonyl Methylglyoxal during Photosynthesis: A Potential Cause of Plant Diabetes — The Calvin Cycle Inevitably Produces Sugar-Derived Reactive Carbonyl Methylglyoxal During Photosynthesis: A Potential Cause of Plant Diabetes — Supplementary Data 

# The Calvin Cycle Inevitably Produces Sugar-Derived Reactive Carbonyl Methylglyoxal During Photosynthesis: A Potential Cause of Plant Diabetes
